# Management of lumbar radiculopathy due to disc herniation with interlaminar epidural steroid injection in the presence of multilevel Tarlov cysts in the neural foramina: A case report

**DOI:** 10.1097/MD.0000000000012389

**Published:** 2018-09-14

**Authors:** Semih Gungor, Asli Ozcan

**Affiliations:** a Division of Pain Medicine, Department of Anesthesiology, Hospital for Special Surgery—Weill Cornell Medicine; b Touro College of Osteopathic Medicine, New York.

**Keywords:** disc herniation, epidural injection, HNP, interlaminar, intralaminar, Tarlov cyst, transforaminal, translaminar, treatment

## Abstract

**Rationale:**

Perineural cysts, commonly referred to as Tarlov cysts, are cerebrospinal fluid-filled dilations between the perineurium and endoneurium typically arising at the junction of posterior and dorsal root ganglia in the neural foramina. This anatomical location is in close proximity to usual needle trajectory during performing transforaminal epidural injection, and therefore presents a potential risk of dural puncture and associated complications.

**Patient concerns:**

Severe lower extremity pain interfering with activities of daily living.

**Diagnoses:**

Lumbar radiculopathy secondary to left-sided L4-5 disc herniation in the presence of multilevel Tarlov cysts in the neural foramina.

**Interventions:**

Posterior interlaminar epidural steroid injection technique was preferred, as opposed to transforaminal approach, to avoid potential risk of dural puncture and associated complications in the presence of multilevel Tarlov cysts in the neural foramina.

**Outcomes:**

The patient responded favorably to epidural steroid injection via interlaminar approach with complete resolution of pain, symptoms, and signs. There were no complications. The patient was able to tolerate physical therapy, wean pain medications, and achieve normal activities of daily living without any significant limitations.

**Lessons:**

In patients presenting with an MRI report of “Tarlov cysts”, meticulous evaluation of diagnostic images should be an essential first step before considering invasive spinal procedures. Should there be any presence of Tarlov cyst in close proximity to planned needle trajectory, we recommend appropriate modification of spinal intervention to avoid potential complications.

## 1 Introduction

Perineural cysts were first described by neurosurgeon I.M. Tarlov in 1938 during dissection of cadavers at the Montreal Neurological Institute.^[1]^ Now commonly referred to as Tarlov cysts, these cysts are cerebrospinal fluid (CSF)-filled dilations between the perineurium and endoneurium typically arising at the junction of posterior and dorsal root ganglia.^[2]^ Usually Tarlov cysts are seen in the sacral canal but they can be also found at other lumbosacral levels.^[2]^ Most of the time, these cysts are asymptomatic, discovered as incidental findings on magnetic resonance imaging (MRI) with an estimated prevalence of 5%. However, they can be a cause of low back pain and other radicular symptoms in about 1% of patients, sometimes even leading to bladder and sexual dysfunction depending on the size and location of the perineural cyst.^[3,4]^ As a result, patients may require treatment for their symptoms, 1 nonsurgical intervention being epidural steroid injections.

We present a management of a case of a patient with diagnosis of lumbar radiculopathy secondary to left-sided L4–5 disc herniation. Posterior interlaminar epidural steroid injection (ILESI) was preferred instead of transforaminal approach (TFESI) due to presence of CSF-filled Tarlov cysts in the neural foramina with close proximity to planned needle trajectory. The aim of this report was to emphasize the importance of reviewing imaging studies prior to performing spinal interventions and possible modification of the intervention to avoid potential complications in high risk patients.

## 2 Case presentation

Data were obtained from retrospective chart review in a single academic hospital for 1 patient presented in this case report. Period of recruitment of this patient was between February 20, 2018 and April 16, 2018. Diagnosis was lumbar radiculopathy secondary to left-sided L4–5 herniated nucleus pulposus (HNP) and stenosis of neural foramina. Evaluation, follow-up, and the spinal interventions were performed by the same fellowship trained physician specialized in interventional pain medicine with 17 years of experience in this field. The authors have obtained written consent to publish this case report from the patients. Institutional Review Board approval was obtained for this case report.

**Figure 1:**
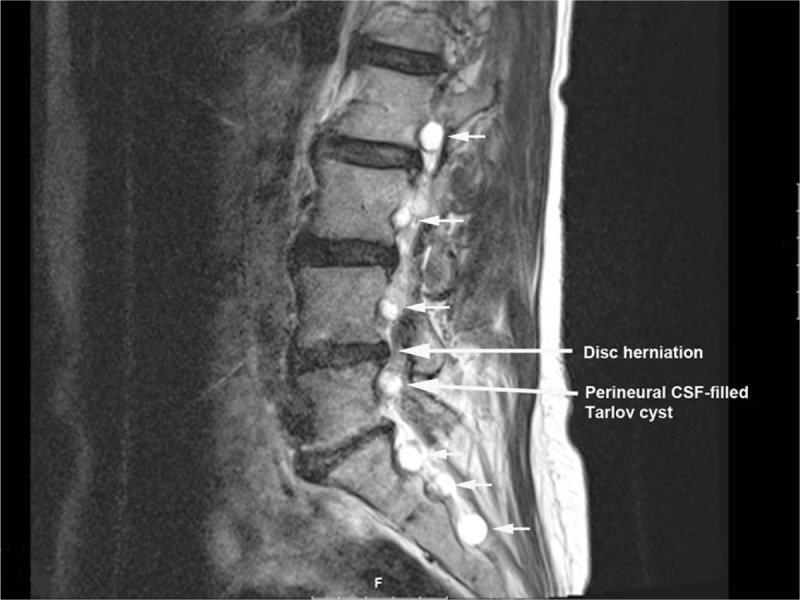
Multilevel foraminal Tarlov cysts (small arrows), in close proximity to disc herniation (large arrow) and Tarlov cysts in close proximity to approximate needle trajectory for transforaminal epidural injection technique (large arrow) on sagittal T2-weighted magnetic resonance imaging.

**Figure 2:**
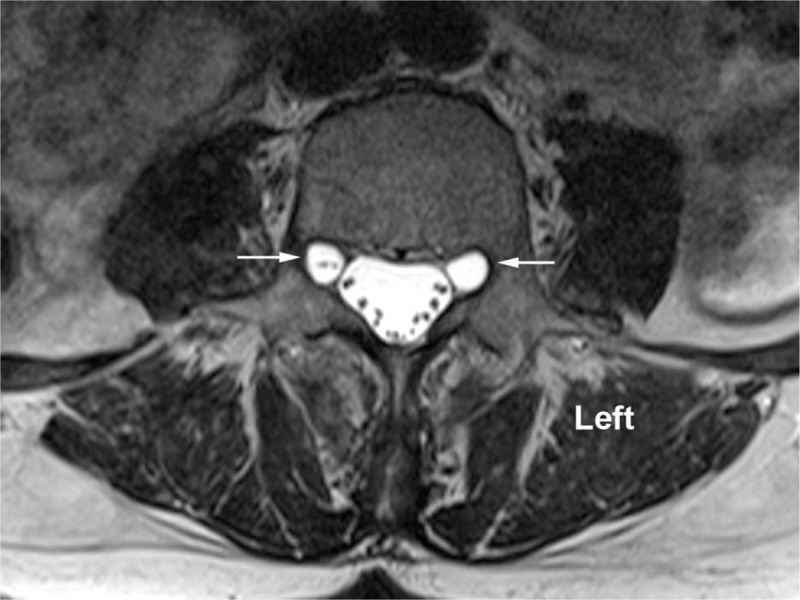
Bilateral foraminal Tarlov cysts at L4–5 level on axial T2-weighted magnetic resonance imaging.

**Figure 3:**
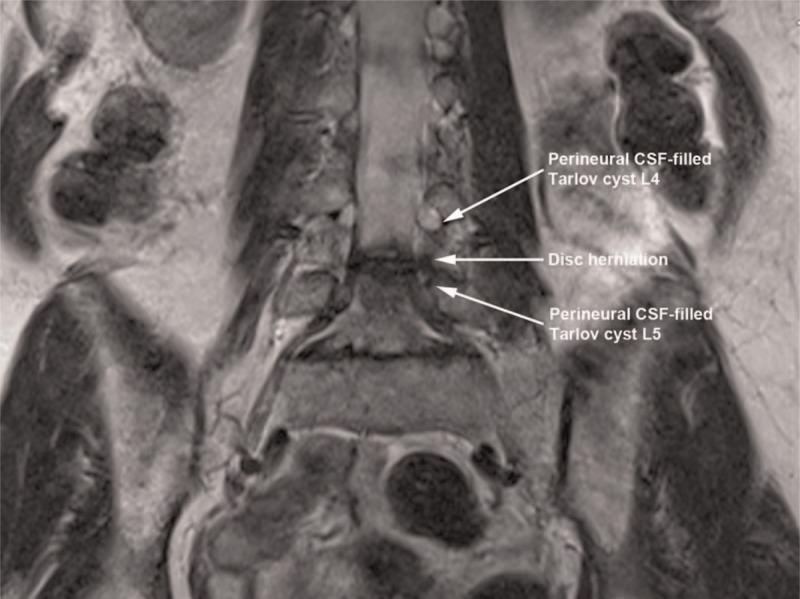
Left-sided disc herniation at L4–5 and foraminal Tarlov cysts in close proximity to approximate needle trajectory for transforaminal epidural injection technique on coronal T2-weighted magnetic resonance imaging.

**Figure 4:**
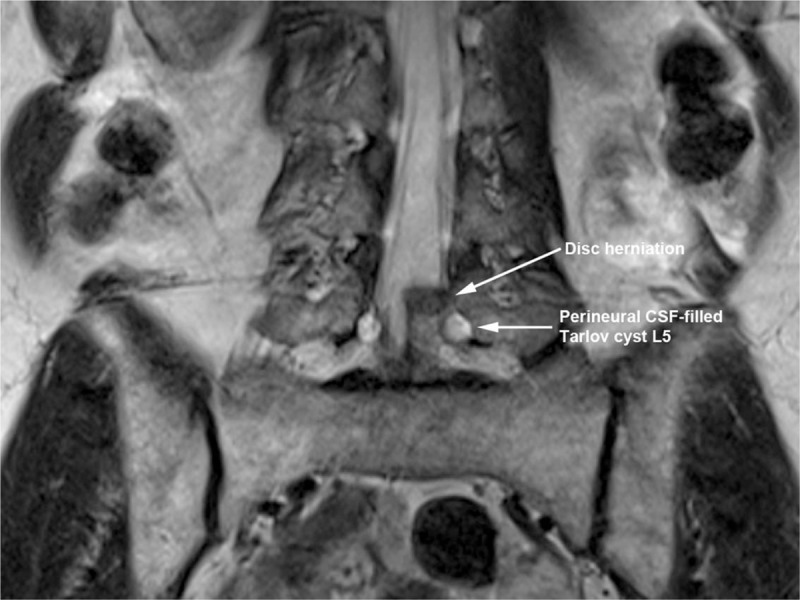
Left-sided disc herniation at L4–5 and foraminal Tarlov cysts in close proximity to approximate needle trajectory for transforaminal epidural injection technique on coronal T2-weighted magnetic resonance imaging.

**Figure 5:**
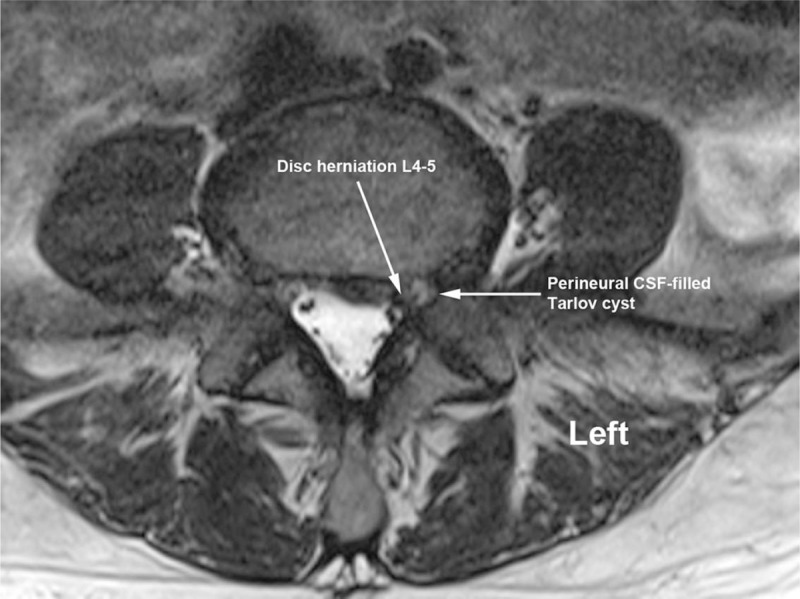
Left-sided disc herniation at L4–5 and foraminal Tarlov cysts in close proximity to approximate needle trajectory for transforaminal epidural injection technique on axial T2-weighted magnetic resonance imaging.

A 64-year-old, otherwise healthy, female presented with a history of gradually increasing left-sided lower back pain and leg pain over 3 months duration. The patient was also complaining of tingling sensation and intermittent numbness in the left foot (in the distribution of Left L4 and L5 nerve roots). Positive findings in the physical examination were limited range of motion of lumbar spine in flexion and left lateral bending secondary to pain, decreased sensation to light touch and pinprick in the distribution of left L4 and L5 nerves and left-sided straight leg raising test being positive with increased left-sided leg pain at 30° in supine position. Deep tendon reflexes were normal and symmetric bilaterally and motor strength was 5/5 in all muscle groups of the lower extremities bilaterally. MRI of lumbar spine showed degenerative changes in the entire lumbar spine, presence of bilateral Tarlov cysts in the T12–L5 neural foramina as well as in the bilateral S1–S3 levels (Figs. 1 and 2). At L4–5 level, there was central and left paracentral HNP resulting in moderate central canal and neural foramen stenosis (Figs. 3–5). At L5–S1 level, there was broad-based disc bulge resulting in mild left-sided neural foramen stenosis. The patient had failed conservative therapy with activity modification, home exercises, medication management including: oral steroids, nonsteroidal anti-inflammatory medications, muscle relaxants, and oxycodone. Physical therapy was started; however, the patient was not able to tolerate the physical therapy secondary to severe pain. As the pain and symptoms were severe and interfering with activities of daily living, epidural steroid injection was planned. Had there been no Tarlov cysts in close proximity to neural foramina of L4–5 and L5–S1 levels, our usual approach would have been left-sided L4–5 and L5–S1 transforaminal epidural approach for steroid injection in a similar patient. However, after reviewing the MRI, we decided to modify our approach to proceed with left interlaminar L5–S1 epidural steroid injection via paramedian approach to avoid possible complications due to injury to Tarlov cysts.

**Figure 6:**
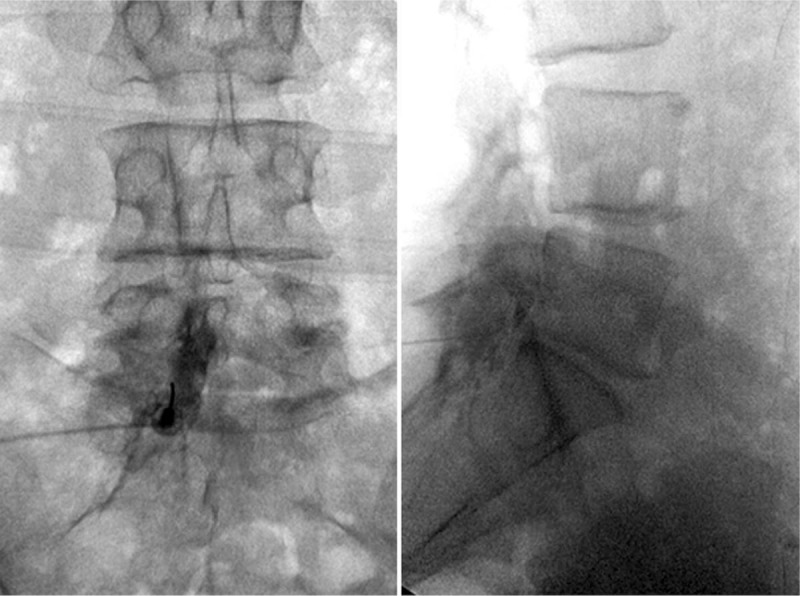
Left-sided interlaminar paramedian epidural injection at L5–S1 level showing anteroposterior and lateral views with contrast confined to area of pathology on the left.

After written informed consent is obtained, the patient is positioned prone on the fluoroscopy table. The back of the patient is prepared with 2% w/v chlorhexidine gluconate in 70% v/v isopropyl alcohol and sterile dressings were applied. Strict sterile technique was adhered to during the entire procedure. Entry site was marked under fluoroscopic view and anesthetized by using 2 mL of lidocaine 1%. Thereafter, by using 22-gauge 3-1/2 inch Touhy needle with loss of resistance to saline technique, left L5–S1 interlaminar epidural space is entered via paramedian approach. After confirming negative aspiration for blood and CSF, 3 mL of iohexol (180 mgI/mL) contrast was injected through the epidural needle, and good distribution of the contrast within the epidural space was verified on anterior–posterior and lateral fluoroscopic views. The contrast was confined to the left side of the epidural space and covering the areas of left L4 and L5 nerve roots (Fig. 6). A 60 mg of triamcinolone acetate mixed with 2 mL of lidocaine 1% is injected through the epidural needle. The patient tolerated the procedure well. There were no complications. On follow-up 10 days after the procedure, the patient was reevaluated. The patient reported 70% relief of the pain and the symptoms. At that stage, the patient was able to resume physical therapy. As the pain and symptoms partially returned 4 weeks after the first procedure, the patient underwent a second epidural steroid injection with a similar interlaminar approach described above. On follow-up 10 days after the procedure, the patient reported more than 90% relief of the pain and symptoms and was not requiring any pain medications. The patient continued physical therapy, and was instructed to continue with home exercises. Within 6 weeks following the first epidural steroid injection, the patient was able to wean all pain medications, walk without any limitation or pain, and was able to return to full-time employment.

## 3 Discussion

Traditionally, epidural steroid injections (ESI) can be performed under fluoroscopic guidance using 2 techniques of administration: interlaminar and transforaminal. In patients with radicular symptoms, the transforaminal approach through the intervertebral foramen is preferred for improved delivery of medications to the targeted anterior epidural space, using less volume of medication.^[5]^ In certain situations, the interlaminar version through the interlaminar space may be utilized.

We present the management of a case where the anatomy is complicated by multiple CSF-filled Tarlov cysts at multiple neural foramina in close proximity to the needle trajectory during transforaminal approach. Therefore, we modified our technique to interlaminar approach as we thought that it would be a safer approach in this particular patient. As discussed earlier, Tarlov cysts contain CSF. Rupture of these cysts can cause CSF leakage which can lead to postdural puncture headache, a widely recognized disabling complication of dural puncture.^[6]^ Furthermore, inadvertent needle insertion into the subarachnoid space increases the risk for serious complications such as aseptic meningitis, bacterial meningitis, and arachnoiditis.^[7]^ Lastly, unintended subarachnoid injection of local anesthetics and steroids have long been reported to have numerous adverse effects such as spinal block and other motor/sensory neurological deficits, seizures, as well as cardiovascular concerns including cardiac arrest.^[8,9]^ In this case, performing ESI using the interlaminar approach theoretically could have reduced the risk of CSF leakage and other complications, while delivering the medications to the area of pathology.

Certain limitations of this case report are single patient, retrospective design, and lack of ability to generalize and establish cause–effect relationship.

## 4 Conclusion

In patients presenting with an MRI report of “Tarlov cysts,” meticulous evaluation of diagnostic images should be an essential first step before considering invasive spinal procedures. Should there be any presence of Tarlov cyst in close proximity to planned needle trajectory, we recommend appropriate modification of spinal intervention. In this case, after the review of anatomy and presence of CSF-filled Tarlov cysts in close proximity to the planned needle trajectory with TFESI, we modified our technique to ILESI in order to avoid the potential risk of dural puncture with transforaminal approach.

## Author contributions

**Conceptualization:** Semih Gungor.

**Data curation:** Semih Gungor, Asli Ozcan.

**Formal analysis:** Semih Gungor, Asli Ozcan.

**Investigation:** Semih Gungor, Asli Ozcan.

**Methodology:** Semih Gungor.

**Resources:** Semih Gungor, Asli Ozcan.

**Software:** Semih Gungor, Asli Ozcan.

**Supervision:** Semih Gungor.

**Validation:** Semih Gungor.

**Visualization:** Semih Gungor, Asli Ozcan.

**Writing – original draft:** Semih Gungor, Asli Ozcan.

**Writing – review & editing:** Semih Gungor, Asli Ozcan.
